# Managing Groundwater Radioactive Contamination at the Daiichi Nuclear Plant

**DOI:** 10.3390/ijerph120708498

**Published:** 2015-07-21

**Authors:** Atsunao Marui, Adrian H. Gallardo

**Affiliations:** 1 Geological Survey of Japan, AIST, Geo-resources and Environment Institute, Groundwater Research Group, Ibaraki-ken, Tsukuba 305-8567, Japan; E-Mail: marui.01@aist.go.jp; 2 CONICET (Argentina National Scientific and Technical Research Council), San Luis National University, FCFMyN, Department of Geology, Ejercito de los Andes 950, San Luis 5700, Argentina

**Keywords:** groundwater contamination, management, radioactivity, control measures

## Abstract

The Great East Japan Earthquake and tsunami of March 2011 severely damaged three reactors at the Fukushima Daiichi nuclear power station, leading to a major release of radiation into the environment. Groundwater flow through these crippled reactors continues to be one of the main causes of contamination and associated transport of radionuclides into the Pacific Ocean. In this context, a number of strategies are being implemented to manage radioactive pollution of the water resources at the nuclear plant site. Along with water treatment and purification, it is critical to restrict the groundwater flow to and from the reactors. Thus, the devised strategies combine walls containment, bores abstraction, infiltration control, and the use of tanks for the temporary storage of contaminated waters. While some of these techniques have been previously applied in other environments, they have never been tested at such a large scale. Therefore, their effectiveness remains to be seen. The present manuscript presents an overview of the methods being currently implemented to manage groundwater contamination and to mitigate the impact of hydrological pathways in the dispersion of radionuclides at Fukushima.

## Communication

The Fukushima Daiichi NPS nuclear meltdown in March 2011 released large amounts of radionuclides that rapidly propagated into the environment. Radiocesium was released into the North Pacific through two major pathways: direct discharge of radioactive water and atmospheric deposition [1]. Needless to say, exposure to radioactively contaminated water and derived food products, pose a serious threat to the populations’ health. Following the accident, the Act on Special Measures was enacted to handle radioactive contamination and emplace an overall policy for decontamination [2]. In line with this policy, a number of strategies have been devised by the nuclear plant operator Tokyo Electric Power Company (TEPCO), national research institutes, and the Japan Atomic Energy Agency (JAEA) in order to contain and/or eliminate the contamination pathways at the site. The pillars of the Fukushima remediation system are measures focused on the storage and treatment of contaminated water, management of waters accumulated in the reactors, interception of groundwater flow to isolate the source of contamination, and development of new technologies to accelerate the site clean-up. None of the works constitute a solution *per se* but, on the contrary, their effectiveness is heavily dependant on the combination of various measures. This approach known as a multilayered containment, has set a number of immediate and long-term goals to reduce radionuclide concentrations and address the contamination problem at the plant. The milestones and targets are regularly reviewed and reassessed based on the progress of implemented strategies. In particular, analyses of all events are collected at the end of each financial year and the implementation of future measures is then determined. Thus, the road map of works is being continuously updated based on achievements and the development of new technologies. 

**Figure 1:**
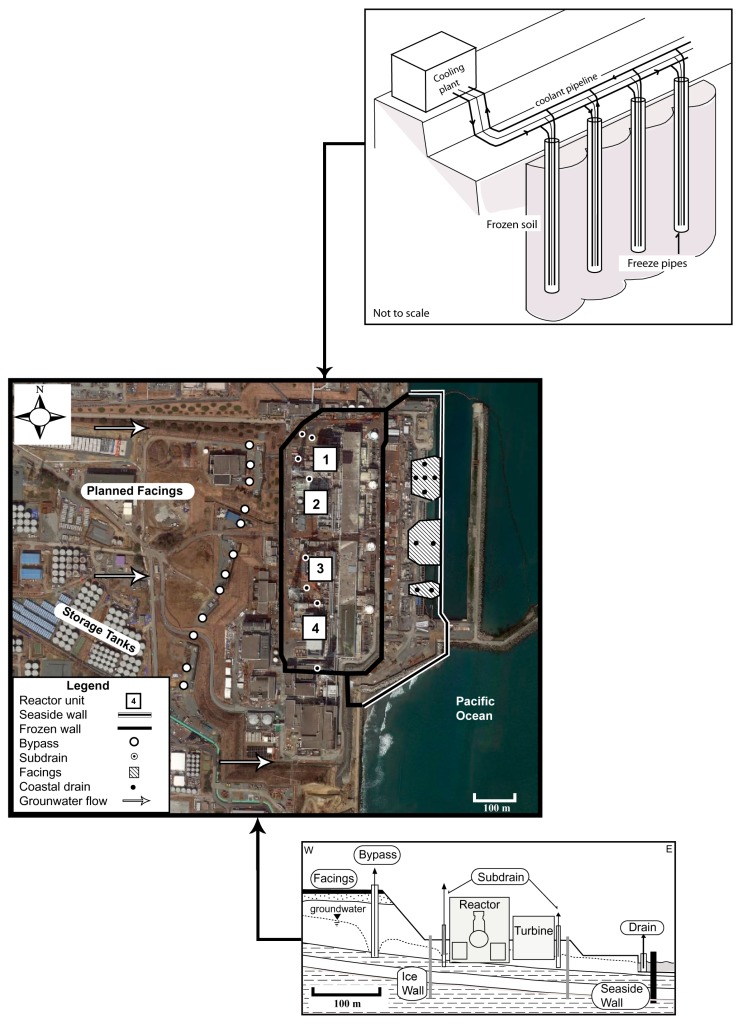
Outline of measures implemented to manage groundwater contamination at the Daiichi nuclear plant.

The Fukushima plant lies on an alluvial terrace within the Hamadori belt, a stretch of Quaternary deposits limited by the Abukuma Granites to the West, and the Pacific Ocean to the East. The geology comprises a sequence of Cainozoic marine and fluvial sediments directly recharged by rain infiltration [3]. Anomalous levels of tritium were measured up to 30 m under the crippled reactors, suggesting a high connectivity between the geological formations. Currently, water accumulated in the reactor buildings is been transferred to storage facilities for treatment on site. More than 1060 tanks are holding up to 1000 m^3^ of water each [4]. Approximately 200,000 m^3^ of contaminated water has been treated so far, whilst an additional 360,000 m^3^ is awaiting processing. Considering that volumes of contaminated groundwater increase at about 400 m^3^/day [5], and the temporary storage is meant to last for no more than three years [6], it is critical to accelerate the construction of additional tanks and expand the water purification capacity. However, those tanks are prone to leakage, have a limited life span, and their number is restricted to the available space. Thus, clean-up works are being complemented with four major strategies to eliminate the source of contamination and reduce the volumes of water to be treated (Figure 1):

- Seaside impervious wall;
- Landward impervious wall (frozen wall);
- Facings;
- Groundwater bypass; subdrain.

The *seaside wall* involves the construction of a steel and concrete wall along the shoreline in front of the reactors (Unit 1 to 4), reaching the base of the aquifers to a depth of 30 m. This wall has been fully completed except for a 10 m section, and is intended to intercept most of the surface runoff and groundwater outflows to the sea. In this regard, pumping bores were also installed at regular intervals to depressurise the soil and prevent the formation of a dam uphill. Collected water is held in tanks and released into the ocean after treatment. Measurements from seawater at the nuclear plant port showed that ^137^Cs levels have decreased from peak concentrations of several thousand Bq/L soon after the accident, to less than 0.55 Bq/L in 2015, well below the guidance level of 10 Bq/L set by the World Health Organization (WHO) for drinking water [7]. These findings would suggest that transport of radionuclides through groundwater might be inhibited by the wall, contributing thus to maintain seawater concentrations within desirable standards. 

A second wall, known as the *ice wall*, is also being constructed around the reactor units to prevent groundwater inflows and their consequent contamination. Simulation studies indicate that inflow to the buildings will gradually decrease until reaching an approximate volume of 30 m^3^/day. The technology consists of enclosing the buildings with a pipe system buried up to about 30 m below the ground, and to circulate a brine refrigerant into them. As a result, the saturated sediments freeze creating a permafrost barrier. Groundwater flowing towards the wall is ultimately diverted to the ocean. The methodology appeared 150 years ago in coal mines in South Wales and it is still widely used in civil and mining engineering [8]. A local-scale trial was successfully started in early 2014, although the sustainability of the *ice wall* remains doubtful. Intensive energy consumption means running costs of several billion dollars a year over several decades. Furthermore, the ice lenses will grow irregularly as per the distribution of chiller pipes, and the sediment desaturation might lead to the aquitards’ compaction and subsidence around the buildings. In effect, a decrease in pore water pressure could increase the effective stress of the ground and result in movements and the formation of fractures in the superficial units. Despite these concerns, the authors argue that, at this stage, there is no compelling evidence to suggest a significant relationship between the ice wall and land subsidence. Furthermore, additional countermeasures, such as re-injection of purified water in vicinities of the wall, could be adopted both to minimise direct discharges to the ocean and to mitigate any potential subsidence throughout the duration of the remediation works. 

Additional measures consist of paving the facilities compound to minimise rainfall infiltration and therefore, reducing recharge to groundwater. *Facings* are being implemented over 1.45 km^2^, starting on the topographic highs behind the reactors, where potentiometric heads and recharge rates are maximum. The effects of the facings would be seen two to three years after completion, but it is predicted that this measure alone could reduce groundwater inflows into the reactors by up to 110 m^3^/day [9]. 

The accumulation of contaminated water in the buildings’ basements is being further reduced by the *underground bypass*. The system comprises 12 bores abstracting an average 300 m^3^/day of water. Their goal is to intercept groundwater on the mountainside of the plant, and redirect it towards the sea upon quality verification. Additional bores (*the subdrain system*) were restored or rebuilt on the sides of the reactor units to locally lower groundwater levels. The plan to discharge into the ocean faced strong opposition from local communities, especially from the fishing industry, which fears a widespread contamination around the already affected areas. Following several months of negotiations, an agreement has been reached to allow for the release of waters with Cs concentrations below 1 Bq/L, and tritium of less than 1500 Bq/L. Up to now, the effects of the *bypass* are unclear, as the general groundwater level dropped only about 20 cm. On the positive side, however, the system helps alleviate the pressure for waste storage, and constitutes a step forward to tackle the contamination as its source.

Once the environment is contaminated, it takes many specialised technologies, and large amounts of resources and time to clean it up [10]. A few years after the tsunami, planning and implementation of recovery works are still ongoing. Addressing the groundwater pollution in Fukushima involves both containing the source and removing the pollutants. Some methods are more widely applied in the clean-up of sites around the world, whilst others technologies are innovative. The effectiveness of these measures remains to be seen. Practical limitations, tight deadlines, and pressure to release land for the displaced residents, pose an unprecedented challenge to all parties. It is anticipated that the described strategies will experience both successes and failures, which are a natural part of the technological advancement, and the lessons learned from Fukushima. 
